# Proactive vs. Reactive Aggression Within Two Modified Versions of the Taylor Aggression Paradigm

**DOI:** 10.3389/fnbeh.2021.749041

**Published:** 2021-09-29

**Authors:** Sara Boccadoro, Lisa Wagels, Alina Theresa Henn, Philippa Hüpen, Lia Graben, Adrian Raine, Irene Neuner

**Affiliations:** ^1^Department of Psychiatry, Psychotherapy and Psychosomatics, Faculty of Medicine, RWTH Aachen University, Aachen, Germany; ^2^JARA-BRAIN Institute Brain Structure and Function, INM-10, Institute of Neuroscience and Medicine, Jülich Research Centre, Jülich, Germany; ^3^Department of Criminology, Psychiatry, and Psychology, University of Pennsylvania, Philadelphia, PA, United States; ^4^Institute of Neuroscience and Medicine 4, INM-4, Jülich Research Centre, Jülich, Germany; ^5^JARA—BRAIN—Translational Medicine, Aachen, Germany

**Keywords:** proactive aggression, reactive aggression, competition, Taylor Aggression Paradigm, skin conductance responses

## Abstract

The Taylor Aggression Paradigm (TAP) has been widely used to measure reactive aggression following provocation during competitive interactions. Besides being reactive, aggression can be goal-directed (proactive aggression). Our study presents a novel paradigm to investigate proactive aggression during competitive interactions. Sixty-seven healthy participants competed in two modified versions of the TAP against an ostensible opponent while skin conductance responses (SCRs) were recorded. During the proactive TAP (pTAP), only the participant could interfere with the ostensible opponent’s performance by blurring the screen. In the reactive TAP (rTAP), the opponent repeatedly provoked the participant by blurring the screen of the participant, impeding their chance to win. In both versions, the blurriness levels chosen by the participant served as a measure of aggression (unprovoked in the pTAP and provoked in the rTAP). In the pTAP, trial-by-trial mixed model analyses revealed higher aggression with higher self-reported selfishness. SCRs decreased with increasing proactive aggression. An interaction effect between gender and proactive aggression for the SCRs revealed increased SCRs at higher aggression levels in females, but lower SCRs at higher aggression levels in males. In the rTAP, SCRs were not associated with reactive aggression but aggression increased with increasing provocation and especially after losing against the opponent when provoked. While males showed higher aggression levels than females when unprovoked, reactive aggression increased more strongly in females with higher provocation. Mean levels of aggression in both tasks showed a high positive correlation. Our results highlight that, despite being intercorrelated and both motivated by selfishness, proactive and reactive aggression are differentially influenced by gender and physiological arousal. Proactive aggression is related to lower physiological arousal, especially in males, with females showing the opposite association. Reactive aggressive behavior is a result of individual responses to provocation, to which females seem to be more sensitive.

## Introduction

Aggression can be classified into two subtypes: *hot-blooded*, reactive aggression, and *cold-blooded*, proactive aggression, two differentially motivated acts that in their extreme form can cause immense harm for the victim. The former type describes impulsive aggressive responses to provocation, while the latter is linked to antisocial behavior and refers to the instrumental and intentional use of aggression to reach a goal (Dodge, 1991; Baron and Richardson, 1994; Dodge et al., 1997). Interestingly, both types may differ not only regarding their motivational source. They are also associated with different physiological, cognitive, and neurobiological mechanisms, including different genetic factors, hormonal influence, and brain circuitry (Wrangham, 2018).

One major physiological difference between both types is the physiological arousal level. In psychological research, skin conductance responses (SCRs) serve as an index of physiological arousal, for individual (state and trait) characteristics of emotional reactions and as an index for direct investigation of stress-related effects on bodily function (Critchley and Nagai, 2013). SCRs measure physiological arousal by detecting changes in sweat secretion by eccrine glands, which are regulated by the autonomic nervous system. Moreover, stressful external or internal stimuli can stimulate sweating (Christopoulos et al., 2019). Prior research linked SCRs to decision-making (Bechara and Damasio, 2005) and demonstrated the utility of SCRs as an indicator of aggression (see meta-analysis by Lorber, 2004). Proactive aggression is associated with low physiological arousal, while reactive aggression is linked to increased physiological arousal (Raine et al., 2006; Armstrong et al., 2019). On the one hand, these differences may go hand in hand with personality traits as low physiological arousal at rest has been reported in groups who show a high rate of cold-blooded violent acts, delinquency, psychopathy, or antisocial behaviors (Gatzke-Kopp et al., 2002; Lorber, 2004). On the other hand, arousal differences may also characterize the psychological state of an individual in situations that promote instrumental aggressive acts in contrast to situations in which aggressive acts are induced via provocation. To our knowledge, most empirical evidence on physiological arousal in situations that promote proactive and reactive aggression comes from studying children. Low SCRs could predict children’s proactive aggression in-the-moment, while high SCRs could predict reactive aggression in-the-moment only at low respiratory sinus arrhythmia, a measure of parasympathetic nervous system activity (Moore et al., 2018). Another group measured SCRs in children playing a board game where they lose against a cheating confederate. The study showed a positive relationship between high SCRs and reactive aggression, but not proactive aggression (Hubbard et al., 2002). In a further study, the authors reported an experiment in which children played three laboratory tasks involving provocation and reward. The results showed that children displayed high SCRs during the two reactive provocation tasks, while displaying low SCRs during the proactive task (Hubbard et al., 2010). Consequently, high physiological arousal associated with state reactive aggression and low physiological arousal associated with state proactive aggression could be assumed. However, laboratory studies supporting this assumption in adults are to our knowledge still lacking.

Furthermore, the association between physiological arousal and aggression might be influenced by gender. While SCRs at baseline in boys with conduct problems and high aggression reported by parents did not differ from those of boys with low aggression, SCRs in aggressive girls with conduct problems were higher at baseline compared to non-aggressive girls (Beauchaine et al., 2008). Overall, SCRs decreased at baseline in girls with high aggression and increased in non-aggressive girls. Similarly, gender differences in a university student sample were observed. Under stress, males showed decreased SCRs associated with increased proactive aggression. Increased SCRs, in contrast, were associated with higher reactive aggression. In females, no association between SCRs under stress and either type of aggression emerged. In another study, students showed lower SCRs if they displayed higher proactive aggressive responses in an aggression task (noise administered to the opponent in the unprovoked condition); this negative association was stronger in males compared to females (Bobadilla et al., 2012). Neither males nor females showed a significant association between SCRs and reactive aggression. Thus, evidence from the literature is scarce and contradictory and the association between SCRs, aggression, and gender needs further investigation.

An effective way to study aggressive behavior and its accompanying physiological state is by applying paradigms that simulate real interactions in controlled laboratory settings. The most widely used task in aggression research is the Taylor Aggression Paradigm (TAP, Taylor, 1967) in which participants play a series of reaction time games against an ostensible opponent. Before each trial, both players choose a punishment level for the opponent, which the player who lost the game receives. Originally, the punishment consisted of electric shocks, but many other versions of the TAP with different kinds of punishment modalities have been developed and used, including aversive noise, heat stimuli, and monetary deductions (e.g., Krämer et al., 2007; Weidler et al., 2019). Across different versions of the TAP, the task reliably induce provocation resulting in reactive aggression, as participants select higher levels of punishment after high provocation compared to low provocation (Weidler et al., 2019; Konzok et al., 2020). In contrast, measuring proactive aggression in laboratory paradigms is more complicated because the paradigm has to elicit instrumental aggression without any sort of provocation. Thus, the majority of studies on proactive aggression only used self-reported questionnaires (e.g., Euler et al., 2017; Zhu et al., 2019b; Wang et al., 2020). To measure proactive aggression, a paradigm should capture unprovoked aggressive behaviors, which aim at obtaining a goal such as a reward (instrumental motivation). Some studies used the first unprovoked trials of the TAP as a measure of proactive aggression (Brugman et al., 2015; Dambacher et al., 2015) or the last unprovoked trials of another similar aggression paradigm (Perach-Barzilay et al., 2013). However, using only a few trials does not allow multiple assessment of aggressive behavior, which is fundamental to having sufficient accuracy and reliability to investigate the physiological and cognitive underpinnings of this type of aggression. Other researchers used a pinball game, in which participants could press the tilt button to block the performance of their opponent and win the game (Atkins et al., 1993; Atkins and Stoff, 1993). The major limitation of this type of task is that it requires specialized equipment that complicates the set-up of the experiment. To address these problems, Zhu et al. (2019a) recently developed a paradigm called the Reward-Interference Task (RIT; Zhu et al., 2019a) by taking inspiration from previous aggression tasks, including the TAP. In the RIT, participants play several rounds of an auditory competitive reaction time task against an ostensible opponent. Importantly, participants only have the possibility to interfere with the opponent’s performance via administering a loud noise, thus increasing their likelihood of winning the trial at the expense of the opponent. The opponent does not have this possibility, thus avoiding any kind of provocation to the participant. The number and average intensity of the selected noise level is an index of proactive aggression, as it measures unprovoked aggressive behaviors directed against another person to obtain a reward. By correlating behavior in the task with self-reported measures reported in questionnaires in a Chinese student sample, the authors demonstrated the validity of the RIT in capturing proactive aggression traits as indicated via self-assessments (Zhu et al., 2019a). Replication of these findings is needed to test if the behavior of participants in the task is independent of the game interference modality. To test these assumptions, we applied a modified version of the paradigm using a different interference modality and additionally measure physiological arousal, which might accompany specific inter-individual differences in aggressive behavior.

To prevent confusion, in the following, we operationalize *state aggression* by our TAP outcome variables and *trait aggression* by self-reported questionnaire data. First, we hypothesize that higher state proactive and reactive aggression are associated with higher self-reported trait aggression in the respective questionnaire’s subscales (proactive and reactive) and with global scores of trait aggression. Furthermore, we assume that higher state proactive aggression is correlated with self-reported measures of selfishness. Since trait proactive and reactive aggression are often intercorrelated (Card and Little, 2006; Raine et al., 2006; Polman et al., 2007; Fite et al., 2009), we expect a high positive correlation between state proactive and reactive aggression. Second, we expect to replicate the provocation effect classically found in the TAP within the rTAP: higher aggression following high provocation than low provocation. This serves to assure that the modification of the paradigm (using interference during the performance rather than punishment after winning trials) did not affect the ability to provoke participants, a fundamental feature for measuring reactive aggression. Third, based on a meta-analysis on gender differences in provoked and unprovoked aggression (Bettencourt and Miller, 1996), we expect higher state proactive and reactive aggression in males compared to females, but the gender difference in reactive aggression to be attenuated by provocation (Weidler et al., 2019). Regarding physiological arousal, we expect a negative association of state proactive aggression (pTAP) with physiological SCRs (amplitudes) and a positive association of state reactive aggression (rTAP) with physiological SCRs. Finally, as the effect of the game outcome (winning and losing) and the belief in the cover story (believing in playing against a real person and not against a computer) are assumed to influence behavior, we are exploring their effect in both paradigms.

## Materials and Methods

As part of a large project, the whole study was preregistered, according to the new gold standard to counteract the replication crisis in psychological research. The pre-registration can be found at https://osf.io/86ezc and includes another task reported elsewhere. Importantly, the trial-by-trial data analyses presented in this manuscript (see section “Data Analysis”) were complementary to the preregistration plan. The method allows for estimation of between- and within-subject variability, thus estimating error terms from the same source, allowing to achieve more power compared to models which average the data. In addition, given that our data were not normally distributed, we assumed a gamma distribution, which is not possible by applying a repeated measures *ANOVA* relying on data that follow a Gaussian distribution. Furthermore it is useful in case of repeated measures, as happens in both paradigms, and allows for the inclusion of cases with missing data (Aarts et al., 2015; Algermissen and Mehler, 2018). Due to our deviation from the preregistration, the mixed-model analysis presented here has an exploratory but not confirmatory character. The data and scripts used in this study are available in the Supplementary Material and at https://github.com/sboccadoro/Proactive-Reactive-Aggression.

### Participants

Seventy healthy adult participants were recruited via flyers, social networks, mailing lists, and contact lists with volunteers from previous studies at the University Hospital RWTH Aachen. Exclusion criteria for participation in the study included a history of psychiatric or neurological illness, first-generation psychiatric illnesses, and insufficient German language skills. Only participants with an age between 18 and 55 years were included. The study was approved by the Ethics Committee of the medical faculty of the University Hospital RWTH Aachen. All subjects provided informed written consent according to the Declaration of Helsinki and were compensated with 30 Euros for their participation.

Due to technical issues during the paradigm recording, one participant was excluded from all analyses. In addition, participants had to be excluded from the SCRs analyses because of problems with physiological arousal measurements (artifacts) in either the pTAP (*n* = 2) and rTAP (*n* = 3). As such, the final sample included 69 participants (mean age = 28.64, *SD* = 10.05, 37 females) for the behavioral analysis. In the context of the SCRs analyses, data from 67 participants (mean age = 28.82, *SD* = 10.14, 36 females) were included for the pTAP, and data from 66 participants (mean age = 28.53, *SD* = 10.04, 36 females) were included for the rTAP.

### Procedure

Participants performed four behavioral tasks while SCRs were recorded. Two of the tasks (the pTAP and rTAP) are the focus of the present study and are described in detail in the next section (see “Paradigm description”). For all subjects, the order of performance of the TAP was identical (first the pTAP and last the rTAP).

To measure SCRs, two electrodes were placed on the middle phalanges of the index and middle finger of the self-reported non-dominant hand of the participants. Participants were told to rest their hand on the desk in the most comfortable position and to avoid any movements. SCRs data were recorded at 5,000 Hz and a direct current excitation voltage of 0.5 V using Brain Vision Recorder (Brain Products GmbH, Gilching, Germany).^1^ During each paradigm, participants were first instructed about the task. Before the actual paradigm implementation, participants had to play four practice trials to exercise and understand how the paradigms work. Before the start of the experiment, we presented examples of how the screen of the opponent would look like in the four different blurriness conditions (see “Paradigm description”). SCRs were recorded continuously during the paradigms. The recording was synchronized with the paradigms sequence via condition-specific triggers sent by the Presentation^®^ software (Version 18.0, Neurobehavioral Systems, Inc., Berkeley, CA).^2^ After completing all tasks, participants filled in questionnaires and were debriefed about the purpose of the study. The total measurement lasted approximately 2.5 h.

### Paradigm Description

#### Proactive Taylor Aggression Paradigm

**FIGURE 1:**
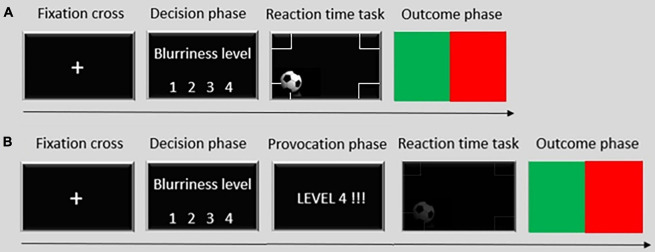
Visual description of **(A)** one trial of the proactive Taylor Aggression Paradigm and **(B)** one trial of the reactive Taylor Aggression Paradigm. *Fixation cross*: participants look at a fixation cross. *Decision phase*: participants have 5 s to select the level of interference for the opponent on a level from 1 (no blurriness) to 4 (maximum blurriness). *Provocation phase* (only in the reactive version of the paradigm): participants see the blurriness level the opponent chose for them. *Reaction time task*: participants play the game, in which they have to press a button as fast as possible when a ball enters any of the target areas at the corners of the field. In the proactive version, they always play at level 1. In the reactive version, they play at the level chosen by the opponent. *Outcome phase*: participants see the outcome of the game represented by a green flash of light if they won and a red flesh of light if they lost. Participants played 40 trials for each task version.

The paradigm was programmed and presented using the Presentation^®^ software of neurobehavioral systems (see text foot note 2). Participants were told that they would compete against an opponent, matched by gender. Due to regulations for the SARS-CoV2 pandemic, participants could not be introduced to an ostensible opponent in person. Thus, to make the cover story believable, fake phone calls were made by the experimenter before the beginning of each paradigm to coordinate with the ostensible experimenter who was measuring the ostensible opponent. Participants were told that they have been pre-assigned to a specific “role” that allows only them (not the opponent) to interfere with the opponent’s performance. Participants played 40 trials of the pTAP. A visual description of the paradigm is available in Figure 1. Each trial started with a fixation cross, followed by the decision phase, in which participants had to choose the level of interference for the opponent on a level from 1 to 4 within 5 s. Level 1 allows the opponent to play with the normal screen visibility. Levels 2, 3, and 4 corresponded to increasing levels of blurriness for the opponent’s screen. During the decision phase, examples of how the screen of the opponent would look like in the four different blurriness conditions were available. There was no limit in the number of times participants could choose each blurriness level. After the decision, participants played the reaction time task, in which they were instructed to press a button as soon as possible when a ball touches any of the four corners at the borders of the screen. After each trial participants saw if they won or lost as indicated by a flash of green or red light, respectively (outcome phase). Participants knew that each trial was worth two euros and that at the end of the whole task, five trials out of 40 would be randomly selected to determine the reward. Thus, winning more trials during the paradigm would correspond to a higher chance of winning more money, with a maximum prize of 10 euros. The task was preprogrammed so that each level of chosen interference was associated with a certain probability of winning the trial. In particular, level one corresponds to a 30% chance of winning the trial, level two to a 50% chance, level three to a 70% chance, and level four to a 90% chance. The whole paradigm duration, including instruction trials, lasted approximately 15 min.

#### Reactive Taylor Aggression Paradigm

The paradigm was programmed and presented using the Presentation^®^ software of neurobehavioral systems (see text foot note 2). Participants were told that they would compete against the same opponent as in the pTAP. The procedure for the cover story (fake phone calls) was the same as in the pTAP. This time, the opponent could interfere with the participants’ performance as well. Participants played a total of 40 trials of the rTAP. A visual description of the paradigm is available in Figure 1. Each trial started with a fixation cross, followed by a decision phase (5 s), in which participants had to choose the level of interference for the opponent on a level from 1 to 4. During the decision phase, examples of how the screen of the opponent would look like in the four different blurriness conditions was available. There was no limit on the number of times participants could choose each level. During the provocation phase, participants saw what level the opponent chose for them. Then participants played the reaction time task at the level of blurriness chosen by the opponent. During the outcome phase, a flash of green or red light indicated that participants either won or lost. Again, participants were told that each trial was worth two euros and that at the end of the whole task, five trials out of 40 were randomly selected representing the reward. The task was preprogrammed and provocation gradually increased during the task. In the first ten trials, participants were mostly unprovoked or provoked at a low level. Every ten trials, the frequency of no provocation and low provocation trials decreased while that of medium and high provocation increased. Again, each level of chosen interference was associated with a certain probability of winning the trial as in the pTAP but adjusted for the level of provocation selected by the opponent, to make the game more believable. The whole paradigm duration, including instruction trials, lasted approximately 15 min.

### Questionnaires

After completing the paradigms, participants were administered a series of questionnaires (German versions) to assess several neuro-psychological variables. As this study was part of a larger project, participants completed a battery of questionnaires. This included the Barratt Impulsivity Scale 11 (BIS-11; Patton et al., 1995), the Reactive-Proactive Aggression Questionnaire (RPQ; Raine et al., 2006), the Buss Perry Aggression Questionnaire (BAPQ; Buss and Perry, 1992), the Selfishness Questionnaire (SQ; Raine and Uh, 2019), the Domain-Specific Risk-Taking Scale (DOSPERT-G; Johnson et al., 2004), the Profile of Mood States Questionnaire (POMS; McNair et al., 1971), the Sensitivity to Punishment/Sensitivity to Reward Questionnaire (SPSRQ; Torrubia et al., 2001), the Wortschatztest (WST; Schmidt and Metzler, 1992) and the Trail Making Test (TMT-A and B; Reitan, 1992). Additionally, participants’ strategy and belief in the cover story for the pTAP and rTAP were assessed with a self-developed questionnaire. All questionnaire data were collected via SoSci Survey.^3^ For the objective of the current study, only data from the RPQ, BPAQ, SQ, SPSRQ, POMS, and belief in the cover story were included in the analyses.

### Data Analysis

#### Basic Statistical Analysis

Descriptive statistics were analyzed using SPSS 25.0 software. We calculated mean levels of proactive aggression by averaging the blurriness levels chosen in the pTAP across all 40 trials and mean levels of reactive aggression by averaging the blurriness levels chosen in the rTAP across 39 trials from the second trial onward, to exclude the first trial without preceding provocation. We also calculated the mean levels of reactive aggression following provocation levels 1, 2, 3, and 4, by averaging the blurriness levels chosen in the rTAP across trials following provocation levels 1, 2, 3, and 4, respectively. Since the levels of state proactive and reactive aggression were not normally distributed, non-parametric tests were used. To validate the modification of the rTAP, we first tested if the provocation strength affected aggression in a simple model (see preregistration) as similarly performed in previous studies applying the TAP (Weidler et al., 2019; Konzok et al., 2020). A Friedman Test was conducted to compare the mean levels of reactive aggression following each provocation level. Spearman’s rho correlations were conducted to test the relationships between mean levels of proactive and reactive aggression and self-reported measures of aggression (RPQ and BPAQ), selfishness (SQ), and sensitivity to punishment and rewards (the sensitivity to punishment and sensitivity to reward subscales of the SPSRQ, respectively). Specifically, the proactive aggression subscale and the reactive aggression subscale of the RPQ were used for testing correlations with state proactive aggression in the pTAP and state reactive aggression in the rTAP, respectively. Spearman’s rho correlations between mean state proactive and reactive aggression were also tested. To test the mediating effect of trait aggression, the partial correlation between state proactive and reactive aggression was tested with trait proactive (RPQpro subscale) and trait reactive (RPQre subscale) as covariates. Since trait proactive and reactive aggression have been reported to be highly intercorrelated (Card and Little, 2006; Raine et al., 2006; Polman et al., 2007; Fite et al., 2009), we additionally tested the bivariate correlation between the two subscales of the RPQ (proactive and reactive subscales). Since this correlation was significant (*r*_*s*_ = 0.547, *p* < 0.001), we computed the residuals of trait proactive and reactive aggression to obtain measures of “pure” trait proactive aggression independent of reactive aggression and “pure” trait reactive aggression independent of proactive aggression (Raine et al., 2006). Trait reactive aggression was regressed on trait proactive aggression scores and Pearson standardized residuals (mean = 0, *SD* = 1) were saved as “RPQpro_res,” indexing pure trait proactive aggression. Trait proactive aggression was regressed on trait reactive aggression and the residuals were saved as “RPQre_res,” indexing trait reactive aggression. Spearman’s rho correlation between state and trait aggression was repeated using the residuals. Partial correlations between state proactive and reactive aggression were tested again with the residuals of trait proactive and reactive aggression as covariates. To explore whether self-reported levels of anxiety after the paradigms correlate with state aggression in the two paradigms, Spearman’s rho correlations were conducted between mean levels of proactive and reactive aggression and the Profile of Mood States Questionnaire (POMS) anxiety subscale and POMS item 13 (anxiety). To assess whether the belief in the cover story differed by gender, we ran two separate chi-square tests for the pTAP and the rTAP. Lastly, to test whether the employment status of the participants influenced their aggressive behavior in the tasks, Mann-Whitney *U*-tests were conducted to compare mean state proactive and mean state reactive aggression between participants who are currently employed and those who are not. The test was chosen because the pTAP and rTAP mean aggression levels were significantly deviating from a Gaussian distribution as tested by the Shapiro-Wilk test (pTAP: *p* = 0.004; rTAP: *p* = 0.024).

#### Skin Conductance Responses

SCRs data were preprocessed with BrainVision Analyzer by changing the sampling rate to 20 Hz and visually inspecting the data to adjust for movement artifacts. Data were exported to Ledalab (Benedek and Kaernbach, 2010) and analyzed using the Ledalab toolbox (V.3.4.9) based on the recommended standardized procedures, which includes smoothing using the Gauss-method and a window width of 16 samples and data filtering applying a low-pass Butterworth filter with a 2 Hz cutoff (Benedek and Kaernbach, 2010). We performed a continuous decomposition analysis (CDA). This method decomposes the SCRs data into continuous phasic and tonic activity. The CDA includes four steps: (1) non-negative deconvolution of phasic SCRs data resulting in a driver function and a non-negative remainder, (2) estimation of a parameter describing tonic activity, (3) segmentation of the driver and the remainder to identify single impulses by peak detection, and 4) reconstruction of the SCRs data. Our aim was to assess anticipatory SCRs underlying the time course of decisions in proactive and reactive aggression (decision phase). Thus, we selected the time integral of the phasic driver over the entire decision phase for each task as the dependent variable. The time integral of the phasic driver for each task represents cumulative phasic activity and was used as SCRs data for the trial-by-trial mixed model analysis. The response window was 1–5 s after condition presentation (start of the decision phase), and a minimum amplitude criterion of 0.05 ms was used for peak detection.

#### Trial-by-Trial Generalized Linear Mixed-Effects Model Analysis

A trial-by-trial analysis was conducted using RStudio software (RStudio Team, 2020) by fitting a Generalized Linear Mixed-Effects Model with random intercepts for participants using the *lme4* package (Bates et al., 2015). Statistical tests were done using the *lmerTest* package (Kuznetsova et al., 2017). The significance level was set at an alpha level of 5%. *Post hoc* tests for comparison of significant interactions were conducted using the *emmeans* package (Lenth, 2016). State proactive and reactive aggression levels were treated as single data points for each trial instead of computing the average. Two separate analyses were conducted for proactive and reactive aggression. In each analysis, aggression choices for each trial from the second trial onward (either proactive or reactive) were entered as dependent variables. Gender, belief in the cover story, game outcome of the previous trial, and the questionnaires were entered as covariates in both analyses. In the rTAP analysis, we further included provocation as a factor and an interaction term for provocation and gender and provocation and game outcome. The questionnaires of interest included in the model were the SQ (Selfishness Questionnaire), the BPAQ (Buss Perry Aggression Questionnaire), and both subscales of the SPSRQ (Sensitivity to Punishment/Sensitivity to Reward Questionnaire) for both analyses and the proactive and reactive subscales of the RPQ (Reactive-Proactive Aggression Questionnaire) for the pTAP and rTAP analyses, respectively. The questionnaire data were z-transformed for comparability of parameter estimates in the model. Following best practice recommendations (Barr et al., 2013), we specified the maximal random effects structure of our design by including random slopes and intercepts for all predictors if theoretically plausible. We compared the different models with the *anova* command of the lme4 package and selected the model with the best AIC/BIC criteria. For the pTAP, the best model to converge was one with random slopes for trials and game outcome and a random intercept for subjects. For the rTAP, the best model to converge was one with random slopes for trials and a random intercept for subjects.

The statistical models of the aggression analysis were the following:

pTAP_model <− glmer (aggression_choice ∼ game_outcome + belief_cover_story + gender + BPAQ.z + SQ.z + RPQpro.z + SR.z + SP.z + (1 + Trial.z|Code) + (1 + game_outcome|Code), data = pTAP, family = Gamma (link = log), control = glmerControl (optimizer = “Nelder_Mead,” check.conv.grad = .makeCC (“warning,” tol = 2e-1, relTol = NULL), optCtrl = list (maxfun = 2e5)))

rTAP_model <− glmer (aggression_choice ∼ game_outcome + belief_cover_story + gender + provocation + BPAQ.z + SQ.z + RPQre.z + SR.z + SP.z + provocation:gender + game_outcome:provocation + (1 + Trial.z|Code), data = rTAP, family = Gamma (link = log), control = glmerControl (optimizer = “Nelder_Mead,” check.conv.grad = .makeCC (“warning,” tol = 2e-1, relTol = NULL), optCtrl = list (maxfun = 2e5)))

To test if state proactive and reactive aggression can predict skin conductance responses (SCRs), we ran two additional analyses for SCRs in relation to proactive and reactive aggression. SCRs were first transformed by adding one to avoid zero values. In each analysis, transformed SCRs for each trial from the second trial onward were entered as dependent variables. Aggression choices, gender, belief in the cover story, and game outcome were entered as covariates in both analyses. Additionally, interaction terms for aggression choices and gender, belief in the cover story, and game outcome were included in both analyses. Regarding the analysis of SCRs in the rTAP, we further included provocation as a factor and an interaction term for provocation and gender, provocation and aggression choices, and provocation and game outcome. For the pTAP, the best model to converge included random slopes for trials and a random intercept for subjects. For the rTAP, the best model to converge included random slopes for trials and game outcome and a random intercept for subjects. The statistical analyses and *post hoc* tests were conducted in the same way as for the aggression analysis models.

The statistical models of the SCRs analysis were the following:

pTAP_SCRs <− glmer (SCRsshifted ∼ aggression_choice + game_outcome + belief_cover_story + gender + aggression_ choice:belief_cover_story + gender:aggression_choice + aggression_choice:game_outcome + (1 + Trial.z|Code), data = pTAP, family = Gamma (link = log), control = glmerControl (optimizer = “Nelder_Mead,” check.conv.grad = .makeCC (“warning,” tol = 2e-1, relTol = NULL), optCtrl = list (maxfun = 2e5)))

rTAP_SCRs <− glmer (SCRsshifted ∼ aggression_choice + game_outcome + belief_cover_story + gender + provocation + aggression_choice:belief_cover_story + gender:aggression_ choice + aggression_choice:game_outcome + aggression_ choice:provocation + provocation:gender + game_outcome: provocation + (1 + Trial.z|Code) + (1 + game_outcome| Code), data = rTAP, family = Gamma (link = log), control = glmerControl (optimizer = “Nelder_Mead,” check.conv.grad = .makeCC (“warning,” tol = 2e-1, relTol = NULL), optCtrl = list (maxfun = 2e5)))

## Results

### Descriptive Statistics

Table 1 includes information on descriptive statistics. The RPQ, BPAQ, SQ, SPSRQ, and POMS in the current study showed good internal consistency (RPQ, Cronbach’s α = 0.825; BPAQ Cronbach’s α = 0.881; SQ, Cronbach’s α = 0.862; SPSRQ, Cronbach’s α = 0.810; POMS, Cronbach’s α = 0.891).

**TABLE 1:** Descriptive statistics on the sample.

**Variables**	***N***	***M***	***SD***
Age		28.64	10.05
Gender			
Male	32 (46%)		
Years of education		17.20	3.32
Job (Yes)	42 (61%)		
Smoking (Yes)	10 (14%)		
Belief in cover story			
pTAP (Yes)	33 (48%)		
rTAP (Yes)	32 (46%)		
Proactive aggression (pTAP)		2.26	0.90
Reactive aggression (rTAP)		2.39	0.75
Reactive aggression after provocation level 1 (rTAP)		2.10	0.84
Reactive aggression after provocation level 2 (rTAP)		2.31	0.81
Reactive aggression after provocation level 3 (rTAP)		2.50	0.81
Reactive aggression after provocation level 1 (rTAP)		2.71	0.78
RPQ		7.16	4.50
RPQpro		1.09	1.85
RPQre		6.07	3.45
BPAQ*		59.54	14.47
SQ		16.19	9.15
SPSRQ		18.25	6.77
SP		8.00	4.42
SR		10.25	4.46
POMS Subscale: Anxiety		9.14	9.96
POMS item 13 (anxiety)		0.75	1.27

pTAP, proactive Taylor Aggression Paradigm; rTAP, reactive Taylor Aggression Paradigm; RPQpro, proactive aggression subscale of the RPQ; RPQre, reactive aggression subscale of the RPQ; SP, sensitivity to punishment subscale of the SPSRQ; SR, sensitivity to rewards subscale of the SPSRQ; POMS, Profile of Mood States Questionnaire. *The average score of the BPAQ was calculated on 68 participants due to a missing item for one participant.

### Basic Statistical Analysis

**FIGURE 2:**
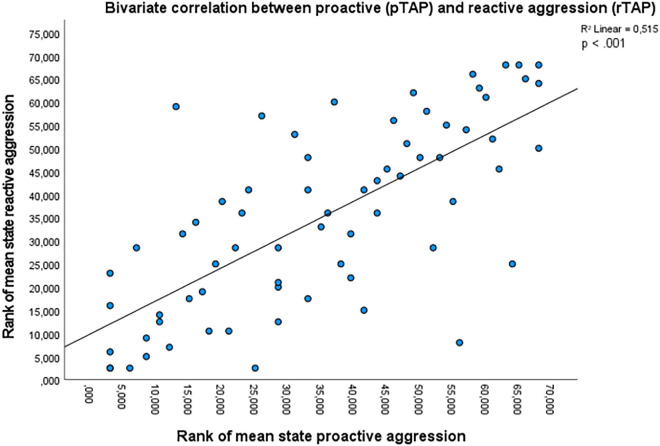
A scatter plot representing the bivariate correlation between the rank of the mean levels of state proactive aggression (pTAP) and of state reactive aggression (rTAP).

A high correlation emerged between mean state proactive and reactive aggression in the two tasks (*r*_*s*_ = 0.718, *p* < 0.001, Figure 2). After controlling for trait proactive and reactive aggression, the correlation remained significant (*partial r_*s*_* = 0.717, *p* < 0.001). The correlation remained significant even after controlling for residualized trait proactive and reactive aggression (*partial r_*s*_* = 0.723, *p* < 0.001). For state proactive aggression, only the correlation with the selfishness score was significant (*r*_*s*_ = 0.250, *p* = 0.039), which, however, did not survive Bonferroni correction for multiple comparison. The other correlations between state proactive aggression and the questionnaires were not significant (RPpro: *r*_*s*_ = 0.014, *p* = 0.911; BPAQ: *r*_*s*_ = 0.134, *p* = 0.275; SP: *r*_*s*_ = −0.040, *p* = 0.742; SR: *r*_*s*_ = 0.015, *p* = 0.901). No significant correlation emerged between mean level of reactive aggression on the rTAP and the questionnaires (RPQre: *r*_*s*_ = 0.094, *p* = 0.444; BPAQ: *r*_*s*_ = 0.141, *p* = 0.253; SQ: *r*_*s*_ = 0.221, *p* = 0.068; SP: *r*_*s*_ = 0.007, *p* = 0.953; SR: *r*_*s*_ = −0.044, *p* = 0.722). The correlation between state aggression and residualized trait aggression was not significant for either proactive (*r_*s*_* = 0.005, *p* = 0.969) nor reactive aggression (*r*_*s*_ = 0.105, *p* = 0.389). No significant correlation emerged between state proactive aggression and the anxiety subscale of the POMS (subscale anxiety; *r*_*s*_ = 0.148, *p* = 0.226) or item 13 (anxiety) of the POMS (*r*_*s*_ = 0.147, *p* = 0.228). Similarly, no significant correlation emerged between state reactive aggression and the anxiety subscale of the POMS (*r*_*s*_ = 0.103, *p* = 0.398) and item 13 (anxiety) of the POMS (*r*_*s*_ = 0.046, *p* = 0.709). The Friedman test indicated a significant difference in mean state reactive aggression in the different provocation conditions [χ^2^(3) = 44.822, *p* < 0.001]. The *post hoc* analyses with Wilcoxon signed-rank tests were conducted with Bonferroni correction (significance level set at *p* < 0.008). Provocation level 2 elicited higher mean state reactive aggression compared to provocation level 1 (*Z* = 3.328, *p* < 0.001). Provocation level 3 elicited higher mean state reactive aggression compared to provocation level 1 (*Z* = 5.021, *p* < 0.001) and to provocation level 2 (*Z* = 3.352, *p* < 0.001). Provocation level 4 elicited higher mean state reactive aggression compared to provocation level 1 (*Z* = 5.110, *p* < 0.001), to provocation level 2 (*Z* = 4.369, *p* < 0.001) and to provocation level 3 (*Z* = 3.258, *p* < 0.001). No association was found between belief in the cover story and gender [pTAP: *X*^2^(1) = 2.550, *p* = 0.110; rTAP: *X*^2^(1) = 1.891, *p* = 0.169]. No significant difference in aggressive behavior in the pTAP and the rTAP emerged between participants with and without a job (pTAP: *U* = 492, *p* = 0.356; rTAP: *U* = 553.5, *p* = 0.868) indicating no effect of the participants’ employment status.

In summary, our results indicate a significant positive correlation between state proactive and state reactive aggression and between state proactive aggression and selfishness scores, but no significant correlation with the other questionnaires. Moreover, provocation efficiently elicited reactive aggression, as aggression increased with increasing provocation.

### Generalized Linear Mixed-Effects Model Analyses

**FIGURE 3:**
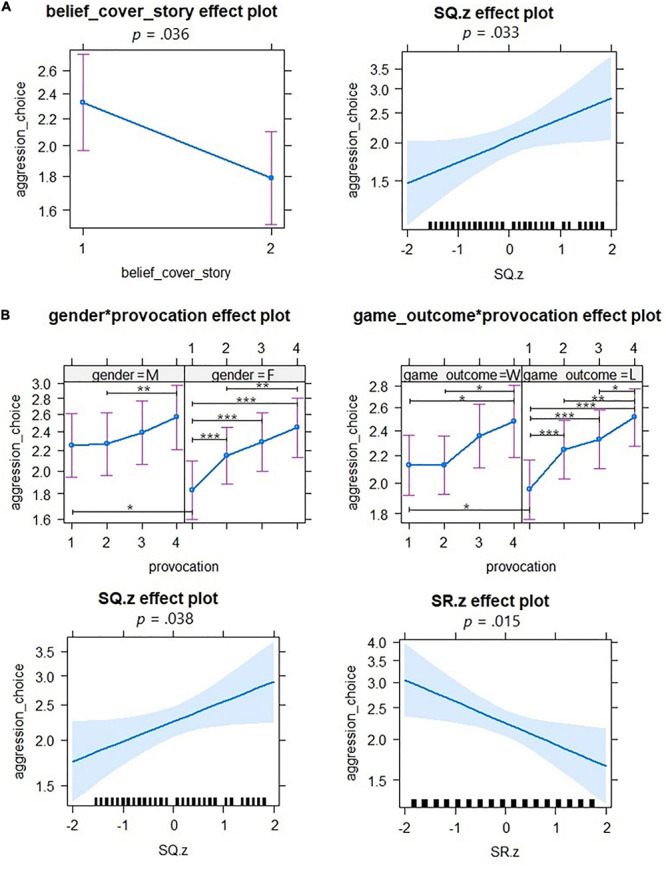
Results of the generalized linear mixed-effects model analyses for the pTAP **(A)** and the rTAP **(B)**. In **(A)**, on the left: main effect of belief in cover story; on the right: main effect of self-reported levels of selfishness (SQ.z). In **(B)**, upper row (left): interaction effect between gender and provocation (M, males; F, females); upper row (right): interaction effect between game outcome and provocation (W, win; L, loss); bottom row (left): main effect of self-reported levels of selfishness (SQ.z); bottom row (right): main effect of self-reported levels of sensitivity to reward (SR.z, SR subscale of the SPSRQ). The thick black lines in the *x*-axis represent the z-transformed scores of the questionnaires (selfishness scores for the SQ.z and sensitivity to reward scores for the SR.z). The error bars depict confidence intervals (95%). *p < 0.05; **p < 0.01; ***p < 0.001.

For the pTAP, the analysis showed significant main effects of belief in the cover story and selfishness scores in the SQ (see Table 2). Aggression levels as indexed by the choices (blurriness levels) in the pTAP were higher if individuals believed in the cover story and with increasing self-reported levels of selfishness (see Table 2 and Figure 3).

**TABLE 2:** Parameter estimates from the generalized linear mixed-effects model analyses for aggression choices in the pTAP and rTAP.

	**Estimate**	**SE**	**t**	***p***
**pTAP**				
Intercept	0.858	0.110	7.771	<0.001
Game_outcome (loss)	0.058	0.030	1.956	0.050
Belief_cover_story (not believed)	–0.263	0.125	–2.101	0.036
Gender (female)	–0.076	0.120	–0.636	0.525
BPAQ.z	0.047	0.080	0.587	0.557
SQ.z	0.162	0.076	2.129	0.033
RPQpro.z	–0.002	0.070	–0.028	0.977
SR.z	–0.114	0.080	–1.425	0.154
SP.z	–0.063	0.063	–1.007	0.314
**rTAP**				
Intercept	0.917	0.098	9.324	<0.001
Game_outcome (loss)	–0.085	0.035	–2.413	0.016
Belief_cover_story (not believed)	–0.093	0.098	–0.957	0.339
Gender (female)	–0.208	0.104	–1.992	0.046
Provocation2	–0.082	0.043	–1.902	0.057
Provocation3	0.011	0.050	0.216	0.829
Provocation4	0.065	0.060	1.082	0.279
BPAQ.z	0.058	0.070	0.834	0.404
SQ.z	0.126	0.061	2.078	0.038
RPQre.z	0.019	0.064	0.293	0.770
SR.z	–0.154	0.063	–2.440	0.015
SP.z	–0.041	0.048	–0.854	0.393
Gender (female):rTAPprovocation2	0.152	0.048	3.189	0.001
Gender (female):rTAPprovocation3	0.167	0.054	3.100	0.002
Gender (female):rTAPprovocation4	0.161	0.058	2.782	0.005
Game_outcome (loss):provocation2	0.138	0.047	2.910	0.004
Game_outcome (loss):provocation3	0.073	0.052	1.408	0.159
Game_outcome (loss):provocation4	0.098	0.059	1.678	0.093

*SE, Standard Error; pTAP, proactive Taylor Aggression Paradigm; rTAP, reactive Taylor Aggression Paradigm; BPAQ.z, z-score of the Buss and Perry Aggression Questionnaire data; SQ.z, z-score of the Selfishness Questionnaire data; RPQpro.z, z-score of the proactive aggression subscale of the Reactive Proactive Aggression Questionnaire data; RPQre.z, z-score of the reactive aggression subscale of the Reactive Proactive Aggression Questionnaire data; SP.z, z-score of the sensitivity to punishment subscale of the Sensitivity to Punishment and Sensitivity to Reward Questionnaire; SR.z, z-score of the sensitivity to reward subscale of the Sensitivity to Punishment and Sensitivity to Reward Questionnaire.*

**FIGURE 4:**
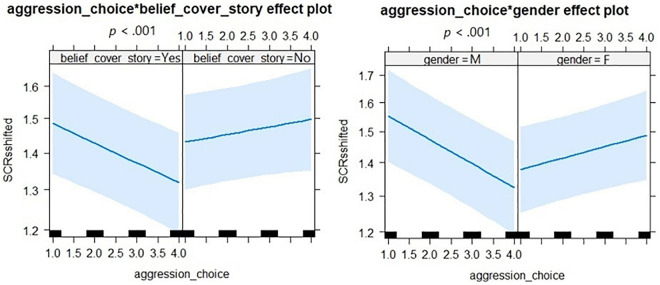
Results of the generalized linear mixed-effects model analyses for SCRs in the pTAP. On the left side: interaction effect between aggression choice and belief in cover story (Yes: believed the cover story, No: did not believe the cover story). On the right: interaction effect between aggression choice and gender (M, males; F, females). SCRsshifted: skin conductance responses values shifted by adding 1. The thick black lines in the *x*-axis represent the aggression choices of the participants (blurriness levels selected). The error bars depict confidence intervals (95%). *p < 0.05.

For the rTAP, the analysis showed a main effect of game outcome, gender, selfishness scores in the SQ and sensitivity to reward in the SPSRQ, and an interaction effect between female gender and levels 2, 3, and 4 of provocation and between losing the previous trial and level 2 of provocation (see Table 2 for statistics of main effects). Aggression levels as indexed by the choices (blurriness levels) in the rTAP were higher in males, after winning the previous trial and with increasing self-reported levels of selfishness. Aggression levels were lower with increasing self-reported levels of sensitivity to reward (Figure 4). *Post hoc* tests for the interaction between gender and provocation revealed that at level 1 (no provocation), males showed higher aggression compared to females (*z* = 1.992, *p* = 0.046). In males, aggression increased only after level 4 of provocation compared to level 2 of provocation (*z* = 3.187, *p* = 0.008). In contrast in females, each level of provocation above 1 elicited increasing aggression compared to level 1, and level of provocation 4 elicited more aggression than level 2 of provocation (for a *post hoc* test on this interaction, see Supplementary Table 1). A *post hoc* test for the interaction between game outcome and provocation revealed that when they were not provoked (level 1) in the previous trial, participants displayed higher aggression after winning compared to losing the previous trial (*z* = 2.413, *p* = 0.016). After winning the previous trial, level 4 of provocation elicited higher aggression compared to levels 1 and 2 of provocation. After losing the previous trial, in contrast, each level of provocation above 1 elicited increasing aggression compared to level 1. In addition, level 4 of provocation elicited higher aggression compared to level 2 and 3 after a loss outcome (for a *post hoc* test on this interaction see Supplementary Table 2).

In summary, in the pTAP aggression increased with belief in the cover story and with increasing selfishness. In the rTAP, aggression was higher in males, with increasing selfishness and with lower sensitivity to reward. Males showed higher aggression than females when unprovoked, while females displayed higher aggression with increasing provocation. Won outcomes increased aggression in the unprovoked and low provocation condition, while loss outcomes increased aggression in all provocation conditions.

For the pTAP, the analysis showed significant main effects of aggression choice and gender and significant interaction effects between aggression choice and not believing the cover story and between aggression choice and female gender. SCRs were lower with increasing aggression levels and in female participants, but higher if individuals did not believe in the cover story. A *post hoc* test for the interaction between aggression choice and belief in the cover story revealed that in participants who believed the cover story, SCRs decreased with increasing aggression, while in participants who did not believe the cover story, SCRs increased with increasing aggression. *Post hoc* tests for the interaction between aggression choice and gender revealed that in males SCRs decreased with increasing aggression, while in females SCRs increased with increasing aggression (see Table 3 and Figure 4). For the rTAP, the analysis only showed a significant interaction effect between provocation (level 4) and game outcome (loss). However, *post hoc* testing did not reveal any significant effect (see Table 3).

**TABLE 3:** Parameter estimates and statistics of the generalized linear mixed-effects model analyses for skin conductance responses in the pTAP and rTAP.

	**Estimate**	**SE**	**t**	***p***
**pTAP_SCRs**				
Intercept	0.562	0.076	7.438	<0.001
Aggression_choice	–0.089	0.016	–5.621	<0.001
Game_outcome (loss)	–0.042	0.027	–1.519	0.129
Belief_cover_story (not believed)	–0.093	0.076	–1.225	0.221
Gender (female)	–0.198	0.076	–2.609	0.009
Aggression_choice:belief_cover _story (not believed)	0.055	0.016	3.545	<0.001
Aggression_choice:gender (female)	0.079	0.016	5.056	<0.001
Aggression_choice:game_ outcome (loss)	0.014	0.011	1.286	0.199
**rTAP_SCRs**				
Intercept	0.290	0.060	4.801	<0.001
Aggression_choice	–0.008	0.016	–0.480	0.631
Game_outcome (loss)	0.041	0.037	1.108	0.268
Belief_cover_story (not believed)	–0.011	0.051	–0.221	0.825
Gender (female)	–0.082	0.054	–1.538	0.124
Provocation2	0.037	0.043	0.865	0.387
Provocation3	0.076	0.048	1.566	0.117
Provocation4	0.058	0.056	1.032	0.302
Aggression_choice:belief_cover_ story (not believed)	0.001	0.012	0.047	0.963
Aggression_choice:gender (female)	0.012	0.012	0.993	0.321
Aggression_choice:game_outcome (loss)	0.004	0.011	0.316	0.752
Aggression_choice:provocation2	–0.012	0.013	–0.904	0.366
Aggression_choice:provocation3	–0.024	0.014	–1.747	0.081
Aggression_choice:provocation4	–0.007	0.014	–0.531	0.595
Gender (female):provocation2	0.023	0.031	0.742	0.458
Gender (female):provocation3	0.017	0.034	0.484	0.623
Gender (female):provocation4	–0.035	0.037	–0.933	0.351
Game_outcome (loss):provocation2	–0.056	0.031	–1.815	0.070
Game_outcome (loss):provocation3	–0.048	0.033	–1.456	0.145
Game_outcome (loss):provocation4	–0.077	0.039	–1.988	0.047

*SE, Standard Error. pTAP, proactive Taylor Aggression Paradigm; rTAP, reactive Taylor Aggression Paradigm; SCRs, Skin Conductance Responses.*

In summary, in the pTAP, higher aggression and female gender were associated with lower SCRs, while not believing the cover story was associated with higher SCRs. Moreover, in males and in participants who believed the cover story SCRs decreased with increasing aggression, while females and participants not believing the cover story showed the opposite effect. In the rTAP, no significant SCRs effect emerged.

## Discussion

Based on the classical distinction between proactive and reactive aggression (Dodge, 1991; Baron and Richardson, 1994; Dodge et al., 1997), two different tasks have been designed in the current study to measure these two types of aggression. The pTAP, based on the RIT (Zhu et al., 2019a), was designed to include all characteristics necessary to measure proactive aggression: it elicits the deliberate and unprovoked use of aggression (reflected in blurriness levels for the opponent’s screen that only the participant could select) with an instrumental motivation (winning the game and money). The competition against the opponent including win and loss game outcomes increases the ecological validity of the task, reflecting real life possible results of the aggression act, which can be successful or fail. The rTAP instead was designed to include the provocation component (in the form of blurriness levels of the participant’s screen selected by the opponent) that is fundamental to elicit reactive aggression. In our tasks, as well as in real life, proactive and reactive aggression are intercorrelated (Card and Little, 2006; Raine et al., 2006; Polman et al., 2007; Fite et al., 2009) and cannot be completely separated and related to a single motivation. Nevertheless, our tasks constitute a valuable tool to measure the main motivational component leading to proactive and reactive aggression, namely reward-seeking and emotional spiteful motivations, respectively (Runions et al., 2018). Aiming to validate the pTAP, and the rTAP, the current study showed that both versions elicit different behaviors depending on trait factors (i.e., personality variables and gender) and state factors (i.e., provocation or game outcome and belief in the cover story). Interestingly, physiological arousal was specific to proactive aggression, with a missing link during the reactive aggression task. Results from the pTAP indicated a negative association between proactive aggression and SCRs. In line with our findings, evidence in the literature has linked proactive aggression to low physiological arousal (Hubbard et al., 2010; Bobadilla et al., 2012; Moore et al., 2018; Armstrong et al., 2019). Additionally, as similarly indicated in a few studies of young students, the arousal effect in proactive aggression here seems to be gender-specific as this link was observed predominantly in males. Previous studies in student samples have observed reduced SCRs under stress with increasing trait proactive aggression in males, but not in females (Armstrong et al., 2019). Others found that the association between low SCRs and high state proactive aggression was stronger in males compared to females (Bobadilla et al., 2012). It is unclear if this effect is age-dependent or specific to low aggressive groups, as a study on adolescents with conduct disorder reported the opposite effect (Beauchaine et al., 2008). While the evidence is too scarce to draw any firm conclusion, our findings in combination with previous findings might indicate that developmental stages influence the association between SCRs, aggression, and gender. However, our study as well as the studies including student samples were conducted in healthy individuals, while the studies in adolescent samples included boys and girls with conduct problems. Future longitudinal studies should investigate whether the association between physiological arousal and aggression in laboratory paradigms in males and females changes with development and if there are specific patterns for groups with pathological aggressive behavior.

Our findings indicate that male participants display reduced physiological arousal with increasing proactive aggression choices. Moreover, they show a different pattern in the rTAP, which again, is in line with previous observations in child samples. The missing association between reactive aggression and SCRs observed in this study is in line with null results in a student sample using the TAP (Bobadilla et al., 2012). Other studies in children and young adults have even reported positive, not negative, associations between SCRs and reactive aggression (Hubbard et al., 2002, 2010; Moore et al., 2018; Armstrong et al., 2019). This may indicate that reduced physiological arousal, which characterizes aggression, is specifically related to a certain aggression type, i.e., proactive aggression. Considering the positive link between reactive aggression and SCRs reported in several other studies, it remains unclear whether the missing association found in the present study is due to weak effects and low power or low efficacy of the task. Future studies should implement different versions of the task to assess physiological arousal differences under provocation in large samples to test if the absence of an effect is related to reactive aggression, or instead to a specific paradigm.

Proactive and reactive aggression seem to be promoted by selfish motivation. Selfish behavior is connected to personality traits which are referred to as the *Dark triad*, which in turn is related to different facets of aggression (Jones and Neria, 2015; Deutchman and Sullivan, 2018). It has also been found that selfishness is associated with more utilitarian decision-making (Raine and Uh, 2019) that might be especially relevant for competitive tasks, including monetary gain, and influence how much aggression was applied in the task. Future studies are needed that test if selfish motivation also leads to aggression if the act includes physical and more severe harm against an opponent. Interestingly, state reactive but not proactive aggression showed a negative association with sensitivity to reward. Sensitivity to reward indicates to what extent behaviors are motivated by reward. This surprising finding of lower aggression by individuals who have a high reward motivation, is neither in line with the positive effect of selfishness nor in line with previous studies emphasizing the important role of reward in aggression. For instance, a positive association between sensitivity to reward and trait aggression has been reported (Megías-Robles et al., 2021), as well as associations of reactive aggression and the brain’s reward network (Krämer et al., 2007; Chester and DeWall, 2016). Unlike previous TAP versions, in which punishment is administered only after winning trials, in our version interference can occur in each trial. We thus speculate that the modification of the rTAP, in which it was possible for both participants to manipulate the chance of winning, reduced the motivation for harmful interference as this might be followed by a direct harmful interference of the opponent in the next trial. In a way, this might show de-escalating aggressive behavior in reward-driven individuals in order to keep the potential gain high. Importantly, in our task, both provocation and aggression are administered *during* the game, as they aim at interfering with players’ performance. In sum, the modifications of the rTAP may have increased the goal-directed component of the task to win money, probably influencing the participant’s behavior. Despite the surprising influence of reward motivation, behavioral results in the rTAP replicated the frequently demonstrated positive effect of provocation (e.g., Krämer et al., 2007; Repple et al., 2017; Weidler et al., 2019; Konzok et al., 2020).

Contrary to our hypothesis based on a meta-analysis looking at unprovoked trials in the TAP (Bettencourt and Miller, 1996) we did not find higher state proactive aggression in males compared to females. Some theories pose that females are more generous than males in strategic games (e.g., the dictator game, Senci et al., 2020). However, to our knowledge, no data on gender differences in state proactive aggression measured with a task specifically designed to assess proactive aggression is currently available. We can only speculate that unprovoked aggression in the TAP may also be influenced by status-seeking motives and that this effect varies based on gender. Hormones, such as testosterone, may influence this status-seeking behavior, which has been proposed to be the desire to gain or ensure a higher status (Eisenegger et al., 2010; Wagels et al., 2018). Previous findings indicate that a lower social status is linked with increased aggression (Davis and Reyna, 2015) and that males display higher aggressive behaviors when placed in a lower status position than their rivals (Buades-Rotger et al., 2021). In the pTAP, the opponent has a lower status from the start and cannot inflict harm, so no retaliation is expected following aggressive decisions. Therefore, male participants might not be motivated to display the higher level of aggression that they would display if placed at a lower status than the opponent. Future studies may be needed to test if a status compensation for the opponent increases proactive aggressive behavior in males.

In line with our hypotheses, in the rTAP male participants showed overall higher aggression compared to females, but gender differences were attenuated by provocation, as also reported in previous studies and meta-analyses (Bettencourt and Miller, 1996; Zeichner et al., 2003; Archer, 2004; Weidler et al., 2019). Indeed, males showed higher aggression compared to females only when unprovoked (level 1). As provocation increased, females showed progressively increasing aggression, indicating that provocation is particularly efficient in provoking female participants. The same finding was reported in a previous study (Zeichner et al., 2003; Weidler et al., 2019). In males, only the highest level of provocation elicited higher aggression compared to low provocation. The results might indicate that females are more susceptible to provocation compared to males. However, the higher starting level of aggression in males might contribute to attenuating the effect of provocation. As males are already more aggressive before being provoked, the effect of provocation might result in a lower increase in aggression compared to females due to a ceiling effect (only 4 levels of aggression choice are available). In addition, the effect of provocation in females might be attributed to time effects (exposure to prolonged provocation) rather than exclusively to the intensity of provocation. Our paradigm was programmed to progressively increase provocation over time. Thus, the effect of prolonged exposure to provocation and intensity of provocation cannot be disentangled here. Future studies should address this question by differentiating the intensity and time effects of provocation in eliciting aggression in females.

Game outcome differentially influenced aggressive behavior in the two paradigms. In the pTAP, no significant effect of game outcome emerged, indicating that losing does not constitute an important predictor for proactive aggression. In the rTAP, instead, aggression increased following winning, contrary to the findings of other TAP versions applied before (Wagels et al., 2018; Weidler et al., 2019). Yet, this effect interacted with provocation, demonstrating that the weight of the provocation was different depending on the own game success. Only when unprovoked, participants displayed higher aggression after winning compared to losing. When mildly provoked, loss outcomes significantly increased aggression compared to no provocation. Only after high provocation, aggression increased compared to low provocation regardless of the outcome. Thus, losing the game served as an additional provoking element for reactive aggression, likely inducing frustration, which is in line with previous findings (Weidler et al., 2019).

Considering the influence of belief in the cover story in the pTAP, we speculate that the motivation of participants who did not believe in the cover story decreased as they did not consider their choice as relevant. Similarly, physiological arousal, which increases in non-believers, might indicate uncertainty about the outcome, which cannot be controlled if it is preprogrammed. Since this effect can influence behavior and increase arousal level in the task, we may recommend that future studies willing to use this paradigm include a convincing cover story and assess the participants’ belief in it. In the rTAP, instead, no effect of belief in the cover story emerged, as previously reported (Konzok et al., 2020), suggesting that the provocative nature of the TAP might remain stable independent from the belief in the cover story. This idea, however, requires investigation with a task in which participants are specifically instructed to be playing against a computer.

### Limitations

Loss outcomes in the pTAP might introduce a potential frustration element in the task, which may play a part in contributing to different motivations for the aggressive reactions and containing a potential reactive aggressive element. Consequently, proactive and reactive aggression in our tasks cannot be completely separated. Nevertheless, this reflects what happens in real-life scenarios as well, where aggressive behaviors are mostly not related to a single motivation, but rather involve a combination of different motivations, including rage, revenge, reward, and recreation (Runions et al., 2018). Our tasks are designed to capture the main motivational component of proactive and reactive aggression, which are reward-seeking and emotional spiteful motivations, respectively (Runions et al., 2018). Furthermore, competition with win and loss outcomes is necessary to motivate participants to resort to aggression in order to reach a goal. Despite this limitation, our results indicate that the effect of game outcome in the pTAP might be negligible.

Another limitation is that the interference modality included in the paradigms does not allow for measurement of physical aggression. Developing a pTAP in which participants have to resort to unprovoked physical aggression to reach a goal might be of interest in order to measure a form of aggression more similar to the real-world behavior of violent offenders. In addition, more than half of the participants did not believe the cover story. The reason might be that, due to the pandemic situation, we used fake phone calls rather than introducing a real person to the participants to establish the situation of an ostensible real competition. We found an effect of belief in the cover story in the pTAP. Therefore, the low number of participants believing the cover story might have weakened our findings and it constitutes a limitation of the present study.

The paradigms presented in our study included only four levels of interference (and of provocation in the rTAP) that participants could choose. While having few levels facilitates the task for participants and might thus be useful for application in patient groups, adding more levels of aggression and provocation could reduce the ceiling effect of the paradigm. It may also allow the treatment of provocation as a continuous variable rather than as a factor, reflecting more real-life provocations which vary in intensity on a continuous scale. Moreover, the version of the rTAP included in the current study does not allow to separate between exposure to provocation and provocation intensity. Future studies should investigate whether gender differences in aggression following provocation depend on the intensity or the duration of the provocation. Additionally, we did not collect measurements of trait anxiety. Previous studies indicate an effect of trait anxiety on motivated behavior and on sensitivity to punishment-related stimuli (Crocker et al., 2013; Berchio et al., 2019). While state anxiety measured with the POMS did not show a correlation with state proactive and reactive aggression, a role of trait anxiety in influencing aggressive behavior in our tasks cannot be excluded. Future studies using these paradigms should collect trait anxiety measurements to control for the influence of anxiety on aggressive behaviors. Similarly, we did not collect self-reported levels of stress before and after the tasks. As stress may influence SCRs (Critchley and Nagai, 2013; Christopoulos et al., 2019), collecting information on stress levels would be important to control for the effect of stress on SCRs.

## Conclusion

The newly developed pTAP provides a useful tool to investigate physiological, behavioral, and neural correlates of state proactive aggression in a controlled laboratory setting. As demonstrated by the current results, low physiological arousal accompanies higher proactive but not reactive aggressive behavior in males. Observed gender differences on the physiological and behavioral level provide mixed evidence, with females potentially being more stressed at higher aggression levels in the proactive aggression task and more reactive to provocation in the reactive aggression task. Selfishness seems to be a motivator for both aggression types and in both genders. In the reactive aggression task, punishment decisions are driven by provocation and losing the game serves as an additional provoking element. Thus, gender, physiological arousal and state factors, such as game outcome, differentially influence and distinguish proactive and reactive aggression.

## Data Availability Statement

The original contributions presented in the study are included in the Supplementary Material and are available on github: https://github.com/sboccadoro/Proactive-Reactive-Aggression. Further inquiries can be directed to the corresponding author.

## Ethics Statement

The studies involving human participants were reviewed and approved by the Ethics Committee of the medical faculty of the University Hospital RWTH Aachen. The participants provided their written informed consent to participate in this study.

## Author Contributions

SB, LW, AH, and PH designed the study. SB, AH, and LG collected the data. SB performed data analysis, interpretation, and wrote the manuscript. LW, AH, PH, LG, AR, and IN revised the manuscript and provided critical feedbacks. All authors contributed to and approved the final manuscript version.

## Conflict of Interest

The authors declare that the research was conducted in the absence of any commercial or financial relationships that could be construed as a potential conflict of interest.

## Publisher’s Note

All claims expressed in this article are solely those of the authors and do not necessarily represent those of their affiliated organizations, or those of the publisher, the editors and the reviewers. Any product that may be evaluated in this article, or claim that may be made by its manufacturer, is not guaranteed or endorsed by the publisher.

## Footnotes

1
https://www.brainproducts.com/index.php

2
www.neurobs.com

3
https://www.soscisurvey.de/
