# Supplementary material for: Proactive vs. Reactive Aggression Within Two Modified Versions of the Taylor Aggression Paradigm
Source: Front Behav Neurosci. 2021 Sep 29;15:749041. doi: 10.3389/fnbeh.2021.749041 (PMC8511695; doi:10.3389/fnbeh.2021.749041)
Supplement: Supplementary file 1 [file Data_Sheet_1.docx]

**Supplementary Table 1**. Post hoc test on the interaction for the interaction between gender and provocation in the rTAP.

| Provocation | Estimate | SE | z | *p* |
| --- | --- | --- | --- | --- |
| *Gender = males* |  |  |  |  |
| 1 - 2 | 0.013 | 0.035 | 0.378 | .982 |
| 1 - 3 | -0.047 | 0.040 | -1.182 | .638 |
| 1 - 4 | -0.114 | 0.045 | -2.548 | .053 |
| 2 - 3 | -0.060 | 0.036 | -1.679 | .335 |
| 2 - 4 | -0.128 | 0.040 | -3.187 | .008 |
| 3 - 4 | -0.067 | 0.040 | -1.683 | .333 |
| *Gender = females*  1 - 2 | -0.139 | 0.032 | -4.263 | < .001 |
| 1 - 3 | -0.214 | 0.038 | -5.710 | < .001 |
| 1 - 4 | -0.275 | 0.043 | -6.428 | < .001 |
| 2 - 3 | -0.075 | 0.034 | -2.198 | .124 |
| 2 - 4 | -0.136 | 0.039 | -3.532 | .002 |
| 3 - 4 | -0.061 | 0.038 | -1.599 | .379 |

*Note.* SE, Standard Error.

**Supplementary Table 2**. Post hoc test on the interaction for the interaction between game outcome and provocation in the rTAP.

| Provocation | Estimate | SE | z | *p* |
| --- | --- | --- | --- | --- |
| *Game outcome = win* |  |  |  |  |
| 1 - 2 | 0.006 | 0.035 | 0.172 | .998 |
| 1 - 3 | -0.094 | 0.042 | -2.253 | .109 |
| 1 - 4 | -0.146 | 0.053 | -2.778 | .028 |
| 2 - 3 | -0.100 | 0.040 | -2.539 | .054 |
| 2 - 4 | -0.152 | 0.050 | -3.029 | .013 |
| 3 - 4 | -0.051 | 0.053 | -0.980 | .761 |
| *Game outcome = loss*  1 - 2 | -0.132 | 0.032 | -4.109 | < .001 |
| 1 - 3 | -0.167 | 0.034 | -4.974 | < .001 |
| 1 - 4 | -0.244 | 0.034 | -7.208 | < .001 |
| 2 - 3 | -0.035 | 0.031 | -1.127 | .673 |
| 2 - 4 | -0.112 | 0.030 | -3.689 | .001 |
| 3 - 4 | -0.077 | 0.029 | -2.628 | .043 |

*Note.* SE, Standard Error.
